# A solid pseudopapillary tumor of the pancreas treated with laparoscopic distal pancreatectomy and splenectomy: a case report and review of the literature

**DOI:** 10.1186/1752-1947-4-387

**Published:** 2010-11-29

**Authors:** Athanasios Marinis, Georgios Anastasopoulos, Georgios Polymeneas

**Affiliations:** 1 Second Department of Surgery, Aretaieion University Hospital, 76 Vassilisis, Sofia's Ave, 11528, Athens, Greece

## Abstract

**Introduction:**

Laparoscopic distal pancreatectomy has been described for more than a decade now and has been considered technically feasible, safe, and with reproducible outcomes. It seems to exhibit several benefits of minimally invasive surgery and should be performed in carefully selected patients.

**Case presentation:**

We report the case of a 55-year-old Greek woman with a solid pseudopapillary tumor of the tail of the pancreas. She underwent a laparoscopic distal pancreatectomy and splenectomy. The histopathologic examination finally revealed a cystic-solid pseudopapillary neoplasm of the pancreas. Solid pseudopapillary tumors of the pancreas are rare and affect predominantly young women. These tumors are of unclear pathogenesis and low malignancy, and surgical resection offers an excellent chance for long-term survival.

**Conclusion:**

This case report indicates that in selected centers and for selected patients, laparoscopic distal pancreatectomy is feasible. The benign characteristics of these tumors make them ideal for laparoscopic excision.

## Introduction

Laparoscopic resection of the pancreas was initially described experimentally in the early 1990s [1]. The first laparoscopic pancreatoduodenectomy was reported in 1994 by Gagner *et al. *[2]. Laparoscopic distal pancreatectomy (with or without splenectomy), on the contrary, may be well suited to the laparoscopic approach, is technically easier because no anastomosis is required, and is more widely accepted [3].

Solid pseudopapillary tumors (SPTs) of the pancreas are rare and affect predominantly young women. These tumors are of unclear pathogenesis and low malignancy, and surgical resection offers an excellent chance for long-term survival [4]. The larger review contains 718 well-documented cases and confirms the characteristics of these tumors [4]. Clinically, they appear as slowly growing masses with or without pain; however, these tumors may appear with other rare symptoms [5]. It is not uncommon, though, to arrive at the final diagnosis only by pathology several weeks after the operation [6,7]. The benign characteristics of these tumors make them ideal for laparoscopic excision [8-10].

We present the case of a woman who successfully underwent a laparoscopic distal pancreatectomy and splenectomy for an SPT of the pancreatic tail, and we review the English literature to clarify the safety and efficiency of laparoscopic distal pancreatectomy.

## Case presentation

**Figure 1:**
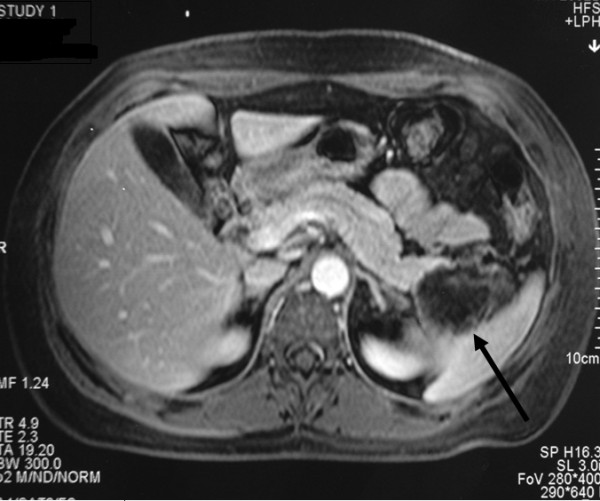
**Magnetic resonance imaging (MRI) scan of the abdomen demonstrating a lesion of the pancreatic tail (arrow) with associated calcifications of the lesion's wall and a central cystic component**.

A 55-year-old Greek woman was referred to our clinic for the management of a cystic lesion located in the tail of the pancreas. The lesion was discovered incidentally during her staging workup with abdominal ultrasound for invasive ductal adenocarcinoma of the left breast 16 months ago. Just after a modified radical left mastectomy had been performed, we further investigated the pancreatic lesion with a magnetic resonance imaging (MRI) scan, which revealed a space-occupying cystic lesion of maximum diameter of 5 cm located in the tail of the pancreas with calcifications of the wall and a central cystic component (Figure 1). Besides the chemo-, radio-, and hormonal therapy she received for her breast cancer, her past medical history also included hypothyroidism under hormone-replacement therapy.

The findings in the physical examination were unremarkable. Blood investigation and tumor markers (CEA, CA 15-3, CA 19-9, and CA 125) were within normal limits. The possibility of a mucinous cystic neoplasm of the pancreas was considered, and a laparoscopic distal pancreatectomy and a splenectomy were chosen.

### Technique

The first distal pancreatectomy in pigs was described by Soper *et al. *[1] in 1994; 2 years later, Gagner [11] reported his first five cases of spleen-preserving laparoscopic distal pancreatectomy for insulinoma.

**Figure 2:**
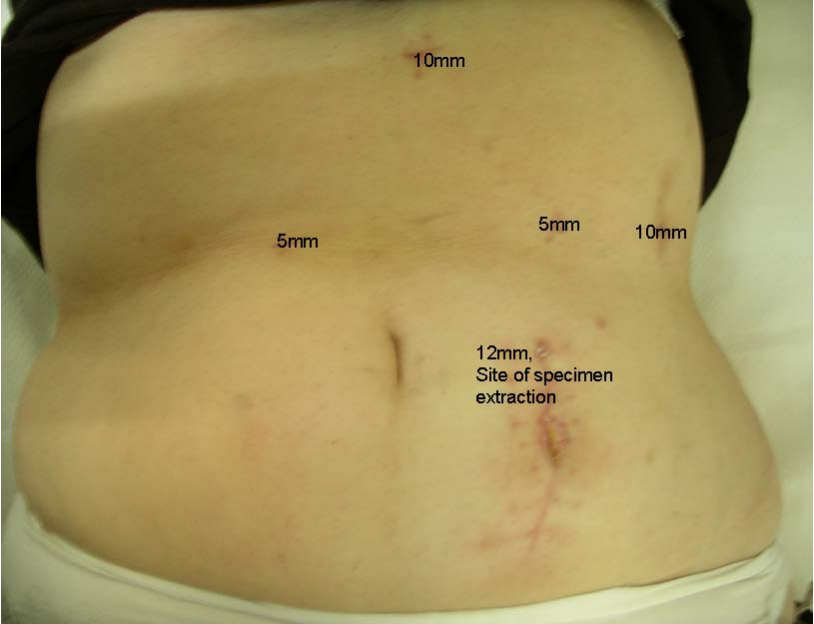
**Postoperative photo showing port entrance wounds**. A five-port technique was used by placing a 12 mm trocar left paramedian at about the level of the umbilicus, a 10 mm trocar in the left upper quadrant of the abdomen on the anterior axillary line, a 10 mm trocar in the subxiphoid region, a 5 mm trocar in the left hypochondrium on the midclavicular line, and a 5 mm trocar in the right hypochondrium in the midclavicular line as well. The specimen was retrieved through a vertical extension of the paramedian port site.

**Figure 3:**
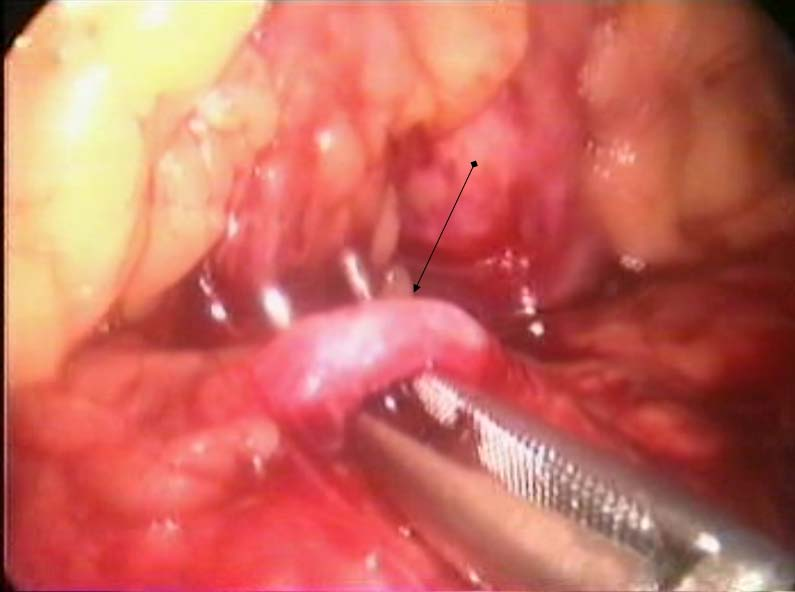
**The splenic artery (arrow) was identified at the upper border of the pancreas and was carefully dissected**.

**Figure 4:**
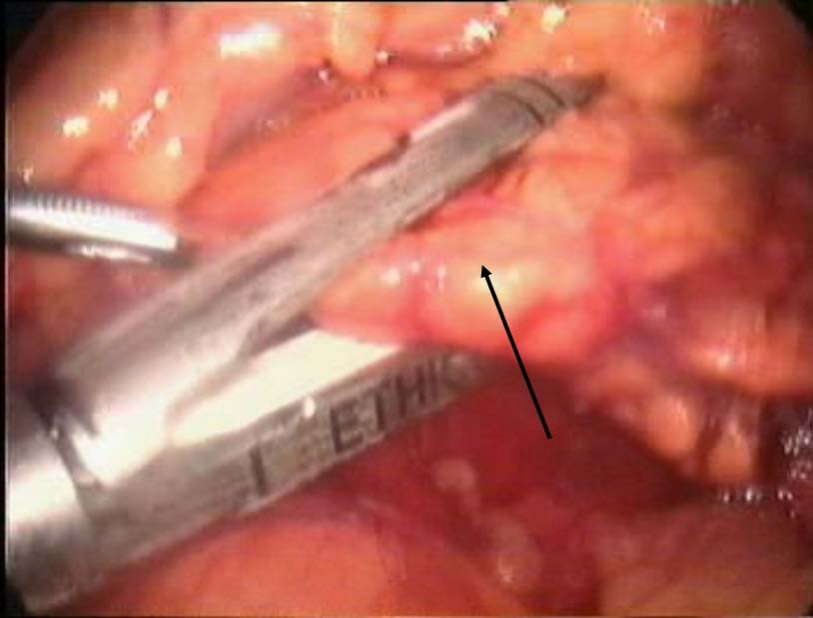
**The pancreas (arrow) was transected by using an endoGIA stapler**.

In our case, the patient was placed in a modified lithotomy position, with the surgeon standing between the legs of the patient. We used a five-port technique, placing a 12 mm trocar left paramedian at about the level of the umbilicus, a 10 mm-trocar in the left upper quadrant of the abdomen on the anterior axillary line, a 10 mm-trocar in the subxiphoid region, a 5 mm-trocar in the left hypochondrium on the midclavicular line, and a 5 mm-trocar in the right hypochondrium in the midclavicular line as well (Figure 2). Pneumoperitoneum was established with the open Hasson technique through the 12 mm paramedian port. Exploratory laparoscopy did not reveal macroscopically evident intra-abdominal metastases. We used a 30-degree laparoscope and an ultrasonic dissector (UltraCision; Ethicon, Endosurgery). After entering the lesser sac, we identified the splenic artery at the upper border of the pancreas after its origin from the celiac axis, and we ligated, by using a disposable clip applicator (U.S. Surgical Corp., Norwalk, CT) (Figure 3). The mobilization of the pancreas started at the reflexion of the superior leaf of the transverse mesocolon on the pancreas. The plane at the inferior border of the body of the pancreas was opened with blunt dissection, gradually exposing the posterior surface of the pancreas. The splenic vein was gently dissected by using a right-angle dissector and was ligated with clips. After sufficiently mobilizing the pancreas, this was transected by using an endoGIA (45 × 2.5 mm) (Figure 4). Subsequent mobilization of the spleen from its attachments to the diaphragm, colon, and left kidney was performed. The specimen was retrieved through a vertical extension of the paramedian port site in a retrieval endobag (Autosuture, Norwalk, CT). A drain was placed in the splenic fossa. Estimated intraoperative blood loss was 320 ml, and no blood transfusion was required. The pathology report revealed a pancreatic tumor 5 cm in diameter. The lesion was multiloculated, contained a yellowish fluid and a thick, stiffened wall, consisting of dense fibrotic tissue with hyaline degeneration, calcifications, regions of ossific metaplasia, and microscopic foci of neoplasmatic tissue, compatible with cystic-solid pseudopapillary neoplasm of the pancreas. Seven reactive regional lymph nodes were harvested, and resection margins were free.

The postoperative course was complicated by a pancreatic fistula (50 ml/d) grade B [12] and a reactive left pleural effusion. On the fifth postoperative day, a CT scan of the abdomen was performed, and a subdiaphragmatic collection was drained under CT guidance. The patient's clinical condition was improved, and the patient was finally discharged on postoperative day 13. The patient's follow-up (with abdominal CT and biochemical tests yearly) in a time period of 36 months revealed no disease recurrence or development of diabetes.

## Discussion

The first use of laparoscopic procedures for the pancreas was in staging of pancreatic cancer in the early 1980s. Subsequently, its use has been widened to palliative procedures for unresectable pancreatic cancer and drainage of pancreatic pseudocysts. About 300 cases of laparoscopic distal pancreatectomy have been reported in the English literature. The largest published series comes from a multicenter European retrospective study, in which 82 cases of laparoscopic distal pancreatectomies from more than 25 institutions are reported [11]. Comparative analysis of the available data reveals several advantages of laparoscopic over open distal pancreatectomy, in terms of wound size, length of hospital stay, time of return to the usual social activities, complication rates, intraoperative blood loss, and the rate of spleen salvage. More specifically, Baker *et al. *[13] and Nakamura *et al. *[14] studied 27 and 21 patients, respectively, who underwent laparoscopic distal pancreatectomies. They concluded that the procedure is a safe, effective modality for managing premalignant neoplasms of the pancreatic body and tail, providing a morbidity rate comparable to that of the open procedure and a substantially shorter length of stay. However, laparoscopic distal pancreatectomy fails to provide a lymphadenectomy comparable to that of open distal pancreatectomy, a fact that may limit the applicability of laparoscopic surgery to the treatment of pancreatic adenocarcinoma.

The issue of spleen preservation is somewhat controversial in the literature and has to do mainly with the underlying pathology. Spleen-preserving distal pancreatectomy may be preferable in the setting of malignant neoplasms, because of its putative mechanism for immune surveillance maintenance. According to Schwartz *et al. *[15], although splenectomy had no significant impact on postoperative recovery after resection of pancreatic adenocarcinoma, it exhibited a negative influence on long-term survival, independent of disease-related factors.

Conversely, Lillemoe *et al. *[16] and Andrén-Sandberg *et al. *[17] recommend that splenectomy should be always performed for oncologic reasons when distal pancreatectomy is performed for cancer. Benoist *et al. *[18] analyzed data from 40 patients who underwent distal pancreatectomy for indications other than chronic pancreatitis and found that distal pancreatectomy with splenectomy had a lower morbidity rate, and pancreas-related complications occurred more frequently after spleen-conserving surgery.

The most suitable lesions amenable to laparoscopic distal pancreatectomy are benign lesions (for example, large serous cystadenomas), chronic pancreatitis, lesions that carry potential for malignant transformation (particularly mucinous cystadenomas and intraductal papillary mucinous neoplasms, IPMNs), and low-grade malignancies such as neuroendocrine tumors and SPTs. Melotti *et al. *[9] reported a series of 58 consecutive patients treated with laparoscopic distal pancreatectomy for solid and cystic pancreatic tumors. They reported no conversions, no mortality, and no intraoperative blood transfusions, and the median hospital stay was 9 days (10.5 days in patients with pancreatic fistula formation). Splenic vessel preservation was feasible in 84.4% of spleen-preserving procedures, and pancreatic fistula formation occurred in 27.5% of all cases.

In our case, the histopathologic examination finally revealed a cystic-solid pseudopapillary neoplasm of the pancreas. This rare neoplasm accounts for 1% to 2% of all exocrine pancreatic tumors, can have either benign or malignant behaviour, and was first described by Frantz in 1959 [19]. SPT is a very uncommon pancreatic tumor that affects mainly women (F/M ratio, 10:1). The mean age at its appearance is 21.97 years (ranging from 2 to 85 years). Most of the patients are young (~22% are younger than 18 years), but a considerable 6% of the patients are older than 51 years [7].

Concerning the surgical approach, many techniques are used. The low grade of malignancy of this tumor has led some surgeons to perform simple enucleation of the neoplasm. However, distal pancreatectomy with splenic preservation or pancreatoduodenectomy, depending on the location of the tumor, represents the procedure of choice. In general, the prognosis, even in the case of a malignant SPT with metastasis, is favorable. Some patients with "unresectable" tumors or those with hepatic metastasis have survived more than 10 years after the operation [7].

## Conclusion

Laparoscopic distal pancreatectomy is feasible in institutions with advanced laparoscopic surgical experience, shares the advantages of minimally invasive surgery, and should be performed in carefully selected patients. SPTs are the ideal pancreatic tumors for the laparoscopic approach because of their low malignancy and their excellent prognosis.

## Consent

Written informed consent was obtained from the patient for publication of this case report and accompanying images. A copy of the written consent is available for review by the Editor-in-Chief of this journal.

## Competing interests

The authors declare that they have no competing interests.

## Authors' contributions

GA analyzed and interpreted the data from the patient's medical file; GA and AM drafted the manuscript; GA, AM, and GP critically revised the manuscript and gave final approval for manuscript publication.
